# Who Cares about Names?

**Published:** 1984-01

**Authors:** 


### WHO CARES ABOUT NAMES?

Our cat Mifanwy has just parted with a furry and appealing ginger litter. She, dear animal, came from a humble background. Born unnoticed amongst a flurry of brothers and sisters on top of a farm haystack in Wales, she was rescued one holiday by our third daughter. The cat's main distinction is a startling ability to climb anything remotely resembling a haystack quicker than you can think of alternatives to the north face of the Eiger. Shortly after introduction to Inner City Life other talents became manifest to the neighbourhood's feline sportsmen of similar inclination.

All anxious parents will guess the result. The last time it was a litter of five in need of good homes. There was no need to worry: a queue developed one Sunday morning, and all the new Celtic pelts were offered homes. As surrogate godparents we were suitably impressed by the literary names to be bestowed upon this urban litter. Horace, Emerson, Norman, Burke and Jane. My!

Alas, the same holidaying daughter soon came home with news of Falls from Grace. Back doorsteps don't lead to such frills. New names now trill the winter evenings. Moggs, Muffin, Theaka (she thinks), Puss-Puss and Tibbs are closer to domestic reality.

What advice to offer? We need it quickly. The 16 year-old male obstetrician of the family has just diagnosed a new pregnancy. Offers of yet more good homes are welcome; we don't care a button about names. Just give us a ring (34294). The kittens are all for free: mother too if you like.

